# Social network types and functional dependency in older adults in Mexico

**DOI:** 10.1186/1471-2458-10-104

**Published:** 2010-02-27

**Authors:** Svetlana Vladislavovna Doubova (Dubova), Ricardo Pérez-Cuevas, Patricia Espinosa-Alarcón, Sergio Flores-Hernández

**Affiliations:** 1 Unidad de Investigación Epidemiológica y en Servicios de Salud Centro Médico Nacional Siglo XXI, Instituto Mexicano del Seguro Social, México; 2 Unidad de Investigación Educativa, Instituto Mexicano del Seguro Social, México; 3 Coordinación de Investigación en Salud, Instituto Mexicano del Seguro Social, México

## Abstract

**Background:**

Social networks play a key role in caring for older adults. A better understanding of the characteristics of different social networks types (TSNs) in a given community provides useful information for designing policies to care for this age group. Therefore this study has three objectives: 1) To derive the TSNs among older adults affiliated with the Mexican Institute of Social Security; 2) To describe the main characteristics of the older adults in each TSN, including the instrumental and economic support they receive and their satisfaction with the network; 3) To determine the association between functional dependency and the type of social network.

**Methods:**

Secondary data analysis of the 2006 Survey of Autonomy and Dependency (N = 3,348). The TSNs were identified using the structural approach and cluster analysis. The association between functional dependency and the TSNs was evaluated with Poisson regression with robust variance analysis in which socio-demographic characteristics, lifestyle and medical history covariates were included.

**Results:**

We identified five TSNs: diverse with community participation (12.1%), diverse without community participation (44.3%); widowed (32.0%); nonfriends-restricted (7.6%); nonfamily-restricted (4.0%). Older adults belonging to widowed and restricted networks showed a higher proportion of dependency, negative self-rated health and depression. Older adults with functional dependency more likely belonged to a widowed network (adjusted prevalence ratio 1.5; 95%CI: 1.1-2.1).

**Conclusion:**

The derived TSNs were similar to those described in developed countries. However, we identified the existence of a diverse network without community participation and a widowed network that have not been previously described. These TSNs and restricted networks represent a potential unmet need of social security affiliates.

## Background

From a health systems perspective, the consequences of the demographic and epidemiological changes of the population have been extensively analyzed. Ageing means an increase in life expectancy, prevalence of chronic diseases, and need for health and social care services [1]. The aging of the population, particularly in developing countries, imposes new challenges and is a field of study that still requires answers from health and social care institutions.

In developed countries, older adults have access to retirement or pension funds, live in nuclear families, receive government and/or community support and social and health care [2]. In contrast in developing countries, older adults (up to 60%) do not receive income from retirement or pension funds, live in extended family groups, rely on family support and receive little support from governmental programs [3].

A topic that is gaining increasing attention is the effect of social networks on the health and wellbeing of older adults. A social network is the collection of interpersonal and communal bonds that people have throughout their lives to establish social relations that satisfy certain needs, and maintain their wellbeing [4]. Social networks can be characterized by their structure, function, and quality. Structure is the number of members, frequency of contacts, variety of links and proximity to the individual. Function comprises the type and frequency of support provided to an individual, and quality is how the individual perceives the structure and function of the network itself [4-6].

The concept of social network type provides a useful framework to derive groups of people with a common set of social ties. There are two prevalent approaches to determine the network type: a) The structural approach [5], which is based on structural components, such as the number of ties and composition, and interactional components, such as the frequency of contacts; b) The multidimensional approach which [6] takes into consideration structural, functional and quality components.

Studies in Europe, Israel, Japan, and the United States have reported that there are different types of social networks (TSNs). The most common are: a) diverse, with distinct sources of potential support (family, friends, neighbours, community groups) and with frequent contact; b) focused on family; c) focused on friends, and; d) restricted in terms of potential sources of support and frequency of contacts [4-7].

The social network patterns and their social relations vary among and within countries, due to cultural and socio-economic conditions [7-10]. For example, Japan and the United States have some unique TSNs, like "married and distal" in the former and structurally and functionally restricted in the latter [7]. TSNs have a differential effect on the health of older adults. Older adults within a restricted network decline in their physical function and overall health status [11-13] and show increased rates of mortality [14-16] and depression [17], whereas individuals related to a diverse network have longer life expectancy and better health status.

The potential effect of social networks is crucial in older adults with high degree of functional, mental or economic dependency. Dependency means that people require social, family or institutional support, due to temporary or definitive loss of their abilities [18]. The prevalence of functional dependency among older adults is high in both, developed and developing countries [19,20]. In México the rates of functional dependency in basic and instrumental activities of daily living (BADL and IADL) is 24% and 23%, respectively [21].

The main risk factors for functional dependency are older age, female sex, low literacy, disease burden, such as cerebrovascular and other chronic diseases, depression, vision and cognitive impairment, lower extremities functional limitation, poor self-perceived health, low level of physical activity, smoking and low frequency of social contacts [19,20,22,23]. Dependency is evaluated and classified in different ways, although in general terms, the greater the dependency, the greater the negative consequences for the older adult, his/her family and for the society.

In developing countries, such as Mexico, most of care for functionally dependent older adults relies on their family and friends, and they can or cannot receive limited institutional support, which comes from social security benefits or social programs. Such support is not universal and it does not necessarily fulfil their social and health needs. To our knowledge, there is information about the characteristics of social networks but there is no information about the types of networks that exist in such countries.

Mexico has 106 million inhabitants and a significant proportion of its population (approximately 48 million) are affiliated with the Mexican Institute of Social Security (IMSS for its acronym in Spanish). Within the population that IMSS covers, 12% are above 60 years of age and 40% of IMSS health care expenditures are allocated to this age group. While a large number of older adults require medical care [24], and do not have information about the types of networks that this age group is part of.

This study has three objectives: 1) To derive the types of social networks among IMSS affiliated older adults; 2) To describe the main characteristics of the older adults in each network type, including the instrumental and economic support they receive and their satisfaction with the network; 3) To determine the association between functional dependency and the type of social network.

## Methods

This study is a secondary data analysis of the 2006 Survey of Autonomy and Dependency (EDAD-IMSS), which was performed on a sample of IMSS affiliated older adults (≥ 60 years old) who are residents of urban areas, without disorders of dementia, according to the Caban Minimental psychometric scale [25].

The survey was conducted to analyze the needs and demands of social services for dependent older adults. The sampling design included four states of the country located in four different geographical regions (North-Durango, West-Nayarit, Central-Mexico City and South Veracruz). These states were selected because of the high proportion of older adults living there.

The sample was stratified by age group and sex since these variables are thought to be related to dependency. A multi-stage sampling approach was performed. In each state, the main city was identified and then the IMSS primary care clinics were located. Next, within the area of coverage of the clinics, a random selection of households in which an IMSS affiliated older adult lived was selected.

To collect the information for the EDAD-IMSS survey, previously trained interviewers carried out direct, structured interviews with the older adults at their homes. The interviewers used a questionnaire on social networks that was previously constructed and applied by the 2004 Guanajuato's State Survey for Older Adults. The EDAD-IMSS survey was approved by the IMSS Research Review Board and IMSS Research Ethical Committee, and older adults provided verbal informed consent.

## Study variables

### Network variables

To derive the TSNs we used the structural approach [5]. The structure and interactional variables included the total number of family members, having a life partner, children, friends, belonging to some community group, and the frequency of contact with family and friends. The total number of family members was obtained by adding all of members who lived under the same roof and outside the older adult's home (children, parents and close relatives) with each other.

Other variables included having a life partner, having children, having friends and belonging to a community group (groups for the elderly, religious, etc.). These were characterized as 0 = no and 1 = yes.

The frequency of personal contacts with friends was measured on a scale ranging from 0 to 5: 0 = no contact, 1 = less than one contact per month, 2 = monthly contact, 3 = contact every two weeks, 4 = weekly contact and 5 = daily contact. The frequency of personal contacts with the family members was obtained by adding the contacts with relatives that live inside (0-5 scale) and outside (0-5 scale) the house of the older adult, therefore, the scale was 0 to 10.

Other network variables included frequency of support received to perform BADL and IADL, which was measured using a scale of 0-5: 0 = absent, 1 = less than monthly frequency, 2 = monthly, 3 = every two weeks, 4 = weekly and 5 = daily. Economic support was interpreted as the monetary and in-kind (groceries, meals, groceries coupons) support that the older adult was receiving from the network members. The categories for economic support were 0 = did not receive economic support, 1 = received economic support. Satisfaction with family and satisfaction with friend's contacts was measured on a scale of 1 to 3, where: 1 = unsatisfied; 2 = more or less satisfied; 3 = totally satisfied.

### Functional dependency and variables to characterize the sample

Functional dependency in basic and/or instrumental activities of daily living (BADL and/or IADL). The BADL were: lying down and getting of the bed, walking from the bed to a chair and vice versa, walking within the house, dressing/undressing, bathing, eating, using the toilet and maintaining sphincter continence. The IADL were using the telephone, shopping, preparing food housekeeping, doing laundry, mode of transportation, responsibility of own medication and ability to handle finances.

To ascertain functional dependency we identified whether the older adult had difficulty to perform BADL and IADL activities by applying the tests of Barthel [26], Katz [27] and Lawton [28]. For the purpose of the analysis, we considered as an independent older adult, a person who did not have limitations in performing BADL activities and had no limitations in up to one IADL activity. A dependent older adult was a person who had limitations in performing one or more BADL and two or more IADL activities. Functional dependency was classified as: 1 = dependent and 0 = independent.

Variables to characterize the sample: a) General characteristics of the older adults such as sex: men and women; age divided into two strata: 60-74 years and ≥ 75 years; schooling; geographic area of residence; 1 = Central (Mexico City), 2 = South (South Veracruz), 3 = West (Nayarit) and 4 = North (Durango).

b) Lifestyles: physical activity classified as 0 = regular (practicing exercise 3 or more times a week, ≥ 30 minutes every time), 1 = irregular or physical inactivity. Diet classified as 0 = healthy diet (daily consumption of fruits, vegetables and dairy products), 1 = unhealthy diet (no consumption or irregular consumption of any of these three groups of food). Smoking: 0 = no (non-smoker during lifetime), 1 = yes (smoker and/or smoked during lifetime).

c) Self-rated health that was measured with the question: In general, would you say your health is: 1) excellent; 2) very good; 3) good; 4) regular 5) poor? The answers allowed classifying the older adults in two types: positive self-rated health = 0, which comprised the categories excellent, very good and good, and negative self-rated health = 1 that included the categories regular and poor.

d) Medical history: number of chronic diseases; depression, which was measured using the Yesavage Geriatric Depression Scale [29] and was classified as 0 = absent, 1 = probable depression, 2 = established depression.

### Statistical analysis

The relative and absolute frequency of categorical variables was determined, as well as the average and standard deviation of continuous variables.

The TSNs were derived from the structure and interactional variables (total number of family members, having a life partner, having children, having friends, belonging to a community group, and frequency of contact with family and friends) by using cluster analysis. Two clustering techniques were applied (hierarchical and non-hierarchical) [6,7]. To carry out the cluster analysis first, the variables were standardized to eliminate the scale difference effects. Then, we performed a hierarchical clustering procedure using the Ward's minimum-variance method and multiple criteria available, such as pseudo-*F *statistics, pseudo-t^2 ^statistic and Sarle's cubic clustering criterion to confirm the ideal number of clusters. Next, a non-hierarchical *k*-means cluster analysis was performed. These techniques allowed identifying the characteristics of homogeneous subgroups to establish the TSNs.

After deriving the network types, we examined the main characteristics of the older adults in each TSN, including the instrumental and economic support they received and their satisfaction with the network. The differences in means of continuous variables among social networks types were compared using one way analysis of variance (ANOVA) followed by a Bonferroni multiple-comparison test, and the differences in proportions for categorical variables among social networks types were compared with the chi-square test.

To evaluate the crude association between functional dependency and the TSN, and the association among dependency and the other covariates, we performed a bivariate analysis that allowed estimating the crude prevalence ratios (PR) and 95% confidence intervals (95% CI).

Due to the controversial results of previous studies regarding the existing differences in the relationship between social networks and functional disability of older adults depending on sex and age, we also determined whether age and sex were confounders or effect-modifiers by applying the stratified Mantel-Haenszel statistics. It was found that these variables were confounders but not effect-modifiers so only analysis for the whole sample was presented.

To obtain the adjusted association between functional dependency (dependent variable) and the TSNs (independent variables), a multiple regression (Poisson regression with robust variance) analysis was performed using the forward selection method. This statistical model estimates directly the PR and 95% CI as better alternative for logistic regression in cross-sectional studies with binary outcomes, because using odds ratio in a cross-sectional study would overestimate the risk ratio (or PR) when the outcome of interest is common (>10%) [30]. A stage entry of variable blocks was used. First, the TSN variables were entered; then, the block of socio-demographic variables and medical history; next the block of lifestyle variables. The variables entered in the model were relevant to the outcome variable, according to the literature [19,20,22,23], and in the bivariate analysis the p value had to be ≤ 0.20. After each entry, the variables within the model were tested for removal based on a p value >0.05.

All analyses were performed with the statistical program Stata (Version 8.1) (Stata; Stata Corp., College Station, TX).

## Results

The survey included 3,426 older adults, from which 64 (1.9%) were excluded because of dementia disorders and 14 (0.4%) answered incompletely. The final analysis included 3, 348 older adults.

**Types of social networks **(Table 1). Five TSNs were identified: diverse network with community participation (12.1%), diverse network without community participation (44.3%); widowed (32.0%); nonfriends-restricted network (7.6%) and nonfamily-restricted network (4.0%).

**Table 1:** Types of social networks

	Diverse with community participation	Diverse without community participation	Widowed	Nonfriends-restricted	Nonfamily-restricted	Total
	**n = 404**	**n = 1484**	**n = 1071**	**n = 255**	**n = 134**	**n = 3348**
Total number of family members, mean (SD)^a^	6.3 (3.0)	6.8 (2.9)	6.3 (3.2)	7.3 (2.9)	1.2 (1.3)	6.4 (3.2)
Having life partner,%	49.8	100.0	0.0	53.3	32.8	55.7
Having children, %	100.0	100.0	100.0	100.0	0.0	96.0
Having friends, %	100.0	100.0	100.0	0.0	93.3	92.1
Belonging to a community group,%	100.0	0.0	0.0	9.2	11.2	13.2
Frequency of contact with family^b^, mean (SD)	7.1 (2.4)	7.9 (1.4)	6.6 (2.6)	6.9 (2.4)	5.8 (2.8)	7.2 (2.2)
Frequency of contact with friends^c^, mean (SD)	3.9 (1.2)	3.5 (1.3)	3.5 (1.4)	0.0 (0.1)	3.3 (1.7)	3.3 (1.6)

^a ^SD - Standard deviation

^b^Frequency of personal contact with family, scale of 0 to 10: sum of contacts with family living inside (0-5) and outside (0-5) of the home of the elderly adult.

^c^Frequency of personal contact with friends, scale of 0 to 5: 0 = no contact, 1 = less than monthly contact, 2 = monthly, 3 = every two weeks, 4 = weekly and 5 = daily.

**The diverse network with community participation **corresponded to a large family network (mean size 6.3 members) in which almost half of the older adults had a life partner, all of them had children and friends and belonged to a community group. The frequency of contact with the family (7.1; SD 2.4) and friends was high (3.9; SD 1.2).

**The diverse network without community participation **was the most frequent. It was composed of the life partner, children or other relatives (mean size 6.8 members) and friends, with whom the older adult had frequent contact. Compared with other TSNs the older adults embedded in this network had the highest average of frequency contact with their family; although they did not belong to a community group.

**The widowed type of network **was the second common network. In this type of network the widowed individuals had children (average family size 6.8 members), with whom they had below average frequency of contacts; furthermore, they had above average frequent contact with their friends. The members of this network did not belong to a community group.

**The nonfriends-restricted network **was characterized by a large family (mean size 7.3 members). In this cluster all older adults had children, half of them had a life partner and they had frequent contacts with their family. The older adults of this group did not have friends and a low percentage (9.2%), were in a community group.

**The nonfamily-restricted network **was characterized by a small number of family members (on average 1.2). Only one third had a life partner and none had children; the frequency of contacts with their family was below average. Most of them had friends (93.3%) with whom they had relatively frequent contact.

**Table **2**shows **the frequency of instrumental, economic support and satisfaction in each type of network. After comparing among the networks, the older adults belonging to the diverse network without community participation and to the nonfriends-restricted network received instrumental support to perform BADL and/or IADL and economic support, more often than those embedded in the other networks. The nonfamily-restricted was the group that received less instrumental and economic support. Across all TSNs, the older adults were satisfied with the contacts they had.

**Table 2:** Frequency of instrumental, economic support and satisfaction in each type of social network

	Diverse with community participation	Diverse without community participation	Widowed	Nonfriends-restricted	Nonfamily-restricted	Total
	**n = 404**	**n = 1484**	**n = 1071**	**n = 255**	**n = 134**	**n = 3348**
^a ^Frequency of instrumental support (to perform BADL and IADL) received,^^† ^mean (SD)	3.3 (2.3)	4.3 (1.7)	3.1 (2.4)	3.7 (2.1)	2.9 (2.4)	3.7 (2.2)
Having received economic support*,%	66.1	74.3	63.6	76.9	41.8	68.8
^b^Satisfaction with family contacts,^^† ^mean (SD)	2.7 (0.7)	2.8 (0.5)	2.7 (0.7)	2.4 (0.9)	2.4 (0.9)	2.7 (0.7)
^b ^Satisfaction with friends contacts^^†^, mean (SD)	2.9 (0.4)	2.8 (0.4)	2.8 (0.5)	0 (0.0)	2.6 (0.8)	2.6 (0.9)

^a ^Frequency of instrumental support received to perform BADL and IADL, scale of 0-5: 0 = absent, 1 = less than monthly frequency, 2 = monthly, 3 = every two weeks, 4 = weekly and 5 = daily

^b ^Satisfaction with family and satisfaction with friend's contacts, scale of 1 to 3: where: 1 = unsatisfied; 2 = more or less satisfied; 3 = totally satisfied

^ *p *< 0.0001, One way analysis of variance

^†^*p *< 0.005, Bonferroni multiple-comparison test

*p < 0.05, Chi-square test

**Socio-demographic characteristics, lifestyle and medical history of older adults in each TSN (Table **3). The diverse with community participation network was most frequent in the southern state of the country; most of its members followed a healthy diet and were classified as functionally independent when compared with the other TSNs. The diverse without community participation network had the highest proportion of men aged <75 years, they reported a higher proportion of regular physical activity and positive self-rated health and had the lower proportion of depression. The widowed network type had relatively more women who had a lower schooling level. The nonfriend-restricted was more common in the central state; its members reported the lowest proportion of irregular and absent physical activity and higher proportions of unhealthy diet, negative self-rated health, depression and functional dependency. The nonfamily-restricted network had the higher proportion of older adults >75 years and it was more frequent in the northern state.

**Table 3:** Socio-demographic characteristics, lifestyle and medical history of older adults in each type of social network

	Diverse with community participation	Diverse without community participation	Widowed	Nonfriends-restricted	Nonfamily-restricted	Total
	**n = 404**	**n = 1484**	**n = 1071**	**n = 255**	**n = 134**	**n = 3348**
**I. Socio-demographic characteristics**	%	%	%	%	%	%
Sex*						
Men	37.9	65.8	28.6	36.1	45.5	47.4
Women	62.1	34.2	71.4	63.9	54.5	52.6
Age*						
<75 years	68.8	74.5	59.2	61.2	51.5	67.0
≥75 years	31.2	25.5	40.8	38.8	48.5	33.0
Schooling*, years, mean (SD)	3.1 (2.3)	3.1 (2.1)	2.7(2.2)	2.5 (2.2)	3.1 (2.1)	2.9 (2.2)
State of residence*						
Central	14.1	19.3	18.8	54.5	14.9	21.0
South	40.8	25.2	28.6	12.2	28.4	27.3
West	27.5	25.0	23.8	18.0	20.9	24.2
North	17.6	30.5	28.8	15.3	35.8	27.5
**II. Lifestyle**						
Physical activity*						
Regular	33.4	34.9	27.7	26.7	32.8	31.7
Irregular and physical inactivity	66.6	65.1	72.3	73.3	67.2	68.3
Diet*						
Healthy diet	80.0	76.5	76.5	58.8	78.4	75.7
Unhealthy diet	20.0	23.5	23.5	41.2	21.6	24.3
Smoking*						
No	69.1	54.7	67.6	63.1	65.7	61.6
Yes	30.9	45.3	32.4	36.9	34.3	38.4
III. Self-rated health*						
Positive	34.9	39.2	33.6	25.5	35.8	35.7
Negative	65.1	60.8	66.4	74.5	64.2	64.3
**IV. Medical history**						
Number of chronic diseases*, mean (SD)	1.3 (1.1)	1.2 (1.1)	1.4 (1.1)	1.4 (1.2)	1.3 (1.1)	1.3 (1.1)
Depression*						
Absent	81.2	84.6	76.0	60.4	79.9	79.4
Probable depression	14.4	12.6	17.1	27.5	14.1	15.5
Established depression	4.4	2.8	6.9	12.1	6.0	5.1
Dependence in basic and instrumental activities*						
Independent	90.1	88.6	82.0	80.4	82.8	85.8
Dependent	9.9	11.4	18.0	19.6	17.2	14.2

*p < 0.05

**Table **4** shows** the crude association between functional dependency, general characteristics, lifestyle and medical history. The bivariate analysis shows that the widowed, nonfriends-restricted and nonfamily-restricted networks were significantly associated with functional dependency. Older adults with dependency in BADL-IADL more likely belonged to these three networks (crude prevalence ratio: 1.8; 1.9 y 1.7 respectively). The analysis of other conceptually relevant variables such as sex, age, education, geographical area of residence, physical activity, diet, self-rated health, number of chronic diseases and depression, shows that they were significantly associated with dependency. Those with higher probability of being dependent were: women, older adults aged 75 years or more, physically inactive or performing irregular physical activity, with negative self-rated health, suffering from more chronic diseases and depression.

**Table 4:** Crude association between functional dependency and type of social network, general characteristics, lifestyle and medical history of older adults (Bivariate analysis)

	Independent n = 2873	Dependent n = 475	Crude Prevalence ratio	Confidence interval at 95%
**I. Type of social network**	%	%		
Diverse with community participation	90.1	9.9	1.0	
Diverse without community participation	88.6	11.4	1.2	0.8 - 1.6
Widowed	82.0	18.0	1.8	1.3 - 2.5
Nonfriends-restricted	80.4	19.6	1.9	1.3 - 2.9
Nonfamily-restricted	82.8	17.2	1.7	1.1 - 2.7
**II. General characteristics**				
**Sex**				
**Men**	88.1	11.9	1.0	
Women	83.8	16.3	1.4	1.2 - 1.6
**Age**				
<75 years	90.0	10.0	1.0	
75 years or more	90.0	10.0	1.0	1.9 - 2.7
Schooling, years, mean (SD)	3.0 (2.2)	2.5 (2.2)	0.9	0.8 - 0.9
State of residence*				
Central	81.7	18.3	1.0	
South	88.0	12.0	0.6	0.5 - 0.8
West	85.8	14.2	0.8	0.6 - 0.9
North	86.8	13.2	0.7	0-5 - 0.9
**III. Lifestyle**				
**Physical activity**				
Regular	93.4	6.6	1.0	
Irregular and physical inactivity	82.3	17.7	2.7	2.1 - 3.4
**Diet**				
Healthy diet	87.3	12.7	1.0	
Unhealthy diet	81.2	18.8	1.5	1.2 -1.8
**Smoking**				
No	85.0	15.0	1.0	
Yes	87.1	12.9	0.9	0.7 -1.0
**IV. Self-rated health**				
Positive	93.6	6.4	1.0	
Negative	81.5	18.5	2.9	2.3 - 3.6
**V. Medical history**				
Number of chronic diseases, mean (SD)	1.2 (1.1)	1.9 (1.2)	1.4	1.4 - 1.5
**Depression**				
Absent	89.9	10.1	1.0	
Probable depression	74.1	25.9	2.6	2.1 - 3.1
Established depression	57.6	42.4	4.2	3.4 - 5.2

**Table **5** describes **the adjusted association between functional dependency and TSNs. After controlling for the confounding and conceptually relevant variables we found that there was a significant association between the widowed network and functional dependency. Older adults with dependency in BADL-IADL more likely belonged to the widowed network (Adjusted Prevalence Ratio: 1.5; 95% CI: 1.1-2.1). There was no association between functional dependency and other network types.

**Table 5:** Adjusted association between functional dependency and type of social network (Multivariate analysis)

	Adjusted Prevalence Ratio	Confidence intervals at 95%
Diverse without community participation	1.3	0.9 - 1.7
Widowed	1.5*	**1.1 - 2.1**
Nonfriends-restricted	1.2	0.9 - 1.8
Nonfamily-restricted	1.5	0.9 - 2.3
Age 75 years or more	1.9*	1.6 - 2.2


Residence		
South	0.8	0.6 - 1.0
West	0.9	0.8 - 1.2
North	1.9*	1.6 - 2.2

Irregular and physical inactivity	1.9*	1.6 - 2.5
Negative self-rated health	1.9*	1.5 - 2.4
Number of chronic diseases	1.2*	1.2 - 1.3


Probable depression	1.9*	1.6 - 2.3
Established depression	2.7*	2.1 - 3.3

*p < 0.05

## Discussion

This study derived a typology of social networks by applying the structural approach that has been used in developed countries [4,5,17]. To our knowledge, previous research of social relationships in México and in other developing countries has been focused on isolated aspects of social relationships [31,32]. Therefore, deriving social network typologies could provide valuable information to better understand the current social conditions of older adults.

Our analysis derived five TSNs among urban dwelling older adults, affiliated with the social security in Mexico: diverse network with community participation, diverse network without community participation, widowed, nonfriends-restricted network and nonfamily-restricted network. These TSNs share similarities, yet they have differences from derived social networks in developed countries.

The diverse network with community participation was characterized by a large network, with many social ties, frequent contacts among members and participation in community groups. The characteristics of this network make it comparable to the diverse networks identified in Israel [4] and the United States [17]. The Mexican diverse network with community participation was larger in size and had more frequent contacts with its family members, than those of the above mentioned countries. In contrast, we derived a type of diverse network without community participation. This network was the most frequent in the present study.

Older adults within diverse social networks (with/without community participation) showed lower proportions of negative self-rated health, depression and dependency. This finding is similar to those from other studies that show that having several sources of support is associated with an improved state of health among older adults [6,13,33,34]. Conversely, people in good states of health tend to have several sources of support.

Belonging to a community group does not replace the support that the family and friends give; although, it has a positive effect. In Mexico, given the results of this study, it seems advisable to create or promote insertion and participation of older adults in community programs. Current social support programs for older adults comprise transfer of economic resources, discounts in public services, and free health services, but these programs do not encourage participation in community groups.

The cluster analysis allowed deriving a widowed network type that has not been previously reported in other studies. The members of this network were predominantly women, they had lost their life partner and they did not belong to any community group. However, all the members of this network had family and friends with whom they had frequent contacts. It seems that the lack of both, life partner and community participation negatively affects older adults. When compared with the diverse networks, the proportion of older adults within the widowed network with negative self-rated health, depression and dependency was higher. Our interpretation is that in Mexico, older adults belonging to the widowed group, who are predominately women, are most vulnerable. Several reasons may account for this situation, mostly due to flawed social policies. For example, in the past women had very low opportunity for social development, such as access to education or employment. The family and eventually the husband were their main providers. Gender equity policies are relatively new and we believe that our results can be a valuable input to design comprehensive interventions for dependent older adults. Further information is needed to ascertain whether widows within this network have the conditions that ease them to have an active relationship with their family and friends and to be involved in community groups.

Two additional types of social networks were derived in the study. The nonfriends-restricted (the older adults have family but no friends) and the nonfamily-restricted (the older adults have friends and a very small number of family members). These types of networks are similar to those reported in the United States, where they are more frequent [17] than in our study. Studies in Europe and Israel have reported only one type of restricted network, which is characterized by limitations in both, family and friend's ties and contacts [4,35].

In accordance with other studies we found that the nonfriends-restricted network had high proportions of negative self-rated health, depression and dependency [17]. The presence of friends in the social network is related to longer life expectancy [36], better self-rated health, lower risk of disability [37] and depression [17]; it has been suggested that friendship may be more influential than family relations on well being [12,38].

The structure and function of social networks are not always correlated; the diverse and restricted networks have both, high and low support [6,7]. Older Mexican adults in diverse networks without community participation and in nonfriends-restricted network received instrumental and economic support more often than those belonging to a diverse with community participations network or to a nonfamily-restricted network. Furthermore, we found that older adults in all TSNs were satisfied with the contacts they had with their family and/or friends. This finding is in accordance with other studies that have reported "a tendency for older adults to report less relationship negativity" [6,39].

In this study, the distribution of the TSNs was different in the states where the survey was conducted. This is probably due to cultural and socio-economic variations, a situation that other countries have reported [8]. Mexico is a mosaic that reflects asymmetry in economic development and cultural differences among the states [40]. The results of the study would serve to develop interventions tailored to the local conditions and considering the predominant type of social network. This would increase the probability of satisfying the needs of specific groups of older adults.

Functionally dependent older adults more likely belonged to a widowed social network. This finding is consistent with previous studies that have stressed that the inability to carry out BADL and IADL increases as the diversity of the social network decreases [13] and greater instrumental support is related to greater risk of functional disability [37,41]. It is important to offer support that stimulates the older adults, while respecting their initiative to carry out activities that they are capable of performing without help, and to identify those for which they need assistance.

In our study, the sex of older adults was not related to dependency for BADL and IADL and to social networks. The results of previous studies are controversial; some have described differences in the relationship between social networks and functionality of older adults depending on sex, while other studies have not confirmed such relationships. Possibly, this is attributable to the convergence of gender roles at these ages in certain cultures [13,42].

Among the limitations of this study, it is worth mentioning that it is a secondary data analysis, which reduces the possibility of an in depth exploration of other aspects of social networks. For example, we did not have information about emotional support. Due to the multidimensional nature of the social networks, having information about such emotional support would enrich the analysis. Also, measuring satisfaction needs further development. The questionnaire addressed satisfaction in general terms and that could overestimate the results. The available information limits the possibility to use a multidimensional approach for the analysis; however, the structured approach provides valuable data to derive the above mentioned types of social networks in the context of Mexican population affiliated to social security. Also, our results are limited to the population of older adults affiliated with the social security and residents of urban areas, whose social networks might be different than those of the rural population or the uninsured. In social security, as well as in urban areas, there are usually more retirees; the labour background might play a role in strengthening the network.

The survey offers cross-sectional information thus it is not possible to make inferences about causal relationships or the direction of the association between the type of network and dependency. The conditions of the TSNs and functional dependency have a dynamic nature, due to changes in different aspects, such as the composition of the social network, in which its members enter and exit continuously; also, the health status of the older adults shows variable periods of dependency and independence. These circumstances create a complex scenario. The scope of this work is limited to the analysis of social networks and functional dependency, although there are other kinds of dependency in the older adult, such as psychological or economic dependency where the relationships between dependency and TSNs might be different.

## Conclusions

We can conclude that this study shows that within the population affiliated with social security, some types of the social networks are similar than those described in developed countries. However, we identify the existence of a diverse network without community participation and of a widowed network that have not been previously described and are frequent. These networks represent a potential unmet need of the social security affiliates.

### Future research and implications

Knowing the type of social network in which the older adult is embedded will facilitate establishing individualized social and health care plans congruent with the older adult social conditions and would increase the potential to design sound and effective social and health care policies aimed at vulnerable older adults. From an academic perspective, it is desirable to carry out longitudinal studies with a multidimensional focus to understand the dynamics of the social networks and their effect on health status.

## Competing interests

The authors declare that they have no competing interests.

## Authors' contributions

SVD and RPC contributed in conceptualizing the research, data analysis and writing the paper. SFH contributed in data analysis. PEA contributed in critically editing the manuscript. All authors participated in the interpretation of data and read and approved the final version to be published.

## Pre-publication history

The pre-publication history for this paper can be accessed here:

http://www.biomedcentral.com/1471-2458/10/104/prepub
